# Natural history of mevalonate kinase deficiency: a literature review

**DOI:** 10.1186/s12969-016-0091-7

**Published:** 2016-05-04

**Authors:** Shumin Zhang

**Affiliations:** Epidemiology, Takeda Development Center Americas, Inc., One Takeda Parkway, Deerfield, IL 60015 USA

**Keywords:** Mevalonate kinase deficiency, Rare diseases, Hereditary autoinflammatory diseases, Hyperimmunoglobulinemia D, Mevalonic aciduria

## Abstract

Mevalonate kinase deficiency (MKD), a very rare autosomal recessive autoinflammatory disease with multiple organ involvement, presents clinically as hyperimmunoglobulinemia D syndrome (HIDS), a less severe phenotype and more common form, and mevalonic aciduria (MVA), a more severe phenotype and rare form. MKD is characterized by recurrent febrile attacks that are frequently accompanied by lymphadenopathy, gastrointestinal symptoms, arthralgia, myalgia, skin rash, and aphthous ulcers. Patients with MVA also have intrauterine growth retardation, congenital defects (cataracts, shortened limbs, and dysmorphic craniofacial features), neurological disease, and failure to thrive. Mean age at onset of symptoms is within the first year of life. There is a delay by several years between symptom onset and diagnosis, which is in part attributable to the initial misdiagnosis due to the rarity and nonspecific clinical manifestations of disease. The frequency of recurrent febrile attacks is highest in childhood and gradually decreases after adolescence. MKD is associated with rare long-term complications such as type AA amyloidosis, joint contractures, abdominal adhesions, renal angiomyolipoma, and severe pneumococcal infections. Frequent febrile attacks significantly impair several aspects of patients’ and caregivers’ quality of life, with an adverse impact on patients’ daily activities, education, and employment. Lifespan is generally normal for HIDS whereas MVA can be fatal in early childhood.

## Background

Mevalonate kinase deficiency (MKD) is a very rare, autosomal recessive autoinflammatory disease with multiple organ involvement [1, 2]. MKD is caused by mutations in the gene encoding mevalonate kinase (MVK) leading to reduced or deficient activity of mevalonate kinase. MKD presents clinically as hyperimmunoglobulinemia D syndrome (HIDS), a less severe phenotype and more common form, and mevalonic aciduria (MVA), a more severe phenotype and rare form [1].

Autoinflammatory diseases like MKD represent an area of high unmet needs as there are no approved treatments for many of these conditions. A disease’s natural history data can shed light on its prevalence, incidence, clinical features, complications, progression, and potential biomarkers. According to the book of *Rare Diseases Epidemiology* [3], a disease’s natural history is “the natural course of a disease from the time immediately prior to its inception, progressing through its presymptomatic phase and different clinical stages to the point where it has ended and the patient is either cured, chronically disabled or dead without external intervention.” Data on the disease’s natural history can also inform the clinical trial design (e.g., patient numbers and types, study duration, and selection of biomarkers and endpoints) [4]. In clinical development for rare diseases, natural history data may also possibly be used as a historical comparator when the observed treatment effect in the clinical trial is considerably greater than variability in disease course [4]. This review summarized the existing literature related to MKD natural history. Search of literature published in English was carried out primarily in PubMed (US National Library of Medicine) using MeSH terms and free text and by checking key references in relevant publications.

### Prevalence and incidence

MKD is considered an ‘ultra-rare’ condition. Worldwide, approximately 300 patients have been reported [1, 5], including approximately 30 patients with MVA [6, 7]. This is likely an underestimate of the population of MKD since some patients may never get diagnosed due to the lack of genetic screening programs for MKD and since not all diagnosed patients may ever be reported to rare disease registries or in the literature [1].

The vast majority of reported patients are European or of European ancestry [2, 8–14], with the largest cluster in the Netherlands and western Europe [1, 14]. About 80 patients with HIDS have been reported from the Netherlands [11], where the founder mutation (p.Val377Ile) is believed to have originated [14, 15]. The Netherlands also has a higher awareness for HIDS, especially with the inclusion of the measurement of immunoglobulin D (IgD) levels in the diagnostic work-up of patients with periodic fever [14]. Only 20 HIDS cases from the United States (US) have been submitted to the international HIDS database [8], which included cases diagnosed up to January 2007 and thus likely underrepresented the current US HIDS patient population.

Data on the prevalence and incidence of MKD are sparse. No published data are available in the US. There are 3 published reports, all in European populations [11, 13, 16], providing the prevalence estimate ranging from 1.3 per 1,000,000 for MKD in Eastern and Central European countries in children aged 0–19 years to 5 per 1,000,000 for HIDS in the whole population in the Netherlands to 6.2 per 1,000,000 for HIDS in Germany in children aged ≤16 years. The incidence of HIDS is also very low with an estimate of 0.39 (95 % confidence interval: 0.22, 0.64) per 1,000,000 person-years in Germany in children aged ≤16 years [16].

### Follow-up studies of natural history

Table 1 summarizes the demographic and clinical characteristics of patients in four largest natural history studies, including the International HIDS Database, a completed, long-term follow-up study of 103 patients with HIDS (excluding patients with MVA) from 18 countries [8], a retrospective French and Belgian chart review of 50 patients with MKD [2], a long-term follow-up of a cohort of 56 patients with MKD in Italy [9], and the Eurofever registry, an ongoing, web-based, international, retrospective follow-up study of autoinflammatory diseases with 114 patients with MKD from 33 countries [5, 10, 17, 18].

**Table 1:** Summary of demographic and clinical characteristics of patients with MKD

Author	van der Hilst et al. [8]	Bader-Meunier et al. [2]		Doglio et al. [9]	Jeyaratnam et al. [17], Toplak et al. [10]^a^, Ter Haar et al. [5]^b^
Study	International HIDS Database	France/Belgium		Italy	International Eurofever Registry
Study design	Retrospective follow-up	Retrospective follow-up		Prospective follow-up	Retrospective follow-up
Study period	1994–2007	1999–2010		N/A	2009–2011^a^
Length of follow-up, years	14	Not specified		Long-term (not specified)	11.5
Number of patients	103 HIDS	50 MKD		56 MKD	114 MKD
Most common MVK mutation (allele frequency in patients)	p.Val377Ile (50 %)	p.Val377Ile (43 %)		p.Val377Ile (47 %)	p.Val377Ile (48 %)
2^nd^ most common MVK mutation (allele frequency in patients)	p.Ile268Thr (15 %)	p.Ile268Thr (8 %)		N/A	p.Ile268Thr (13 %)
Age of patients, years, median or mean ± SD (range)	19 (2, 74)	19.5 (0.6, 58) at last visit		13.3 ± 8.5 at follow-up	14^a^ (1, 60)^a^
% of patients aged <18 years	N/A	N/A		N/A	63 %^a^
Age at onset of symptoms, months, median or mean ± SD (range)	6 (0, 120)	4 (1 day, 240 months)		10.5 ± 15.3 (1, 108)	6 (~0, ~72)^a^
% of patients who had the first attack within the first year of life	78 %	N/A		N/A	71 %^b^
% of patients who had the first attack before the age of 5 years	N/A	92 %		N/A	N/A
Age at diagnosis, years, median (range)	10 (<3 months, 52 years)	N/A		N/A	~8^a^ (~3 months, ~29 years)^a^
Duration from onset to diagnosis, years, median (range)	9.9	N/A		N/A	2.5^a^ (0.1–8.3)^a^
Disease duration, years, mean ± SD (range)	N/A	24 (1, 55) from the onset to most recent assessment		12.4 ± 8.7	13.1^b^ at enrollment
Sex, % of men	50 %	42 %		52 %	46 %
Ethnicity	N/A	White, 69 %		N/A	Caucasian, 90 %
Positive family history, %	N/A	N/A		N/A	26 %^a^
% of patients with >12 fever episodes/year	44 %, aged 0–10 year 24 %, aged 11–20 year 18 %, aged >20 year	76 % at the onset		N/A	N/A
Number of fever episodes per year, mean ± SD (min, max) or median	N/A	N/A		13.8 ± 5.4 (3, 30) at baseline 8.8 ± 6.7 at follow-up	12
Duration of fever episodes, days, mean (range) or median	N/A	3.7 (1, 10)		N/A	5 (3 to 7 in 81 % of the patients)^b^
Precipitating factors of fever episodes, %				N/A	
Vaccination	63 % for the 1st attack	N/A			36 %
Infection	N/A	N/A			17 %
Infection and/or vaccination	N/A	42 %			N/A
Stress	Many cases (not specified)	N/A			24 %
Signs and symptoms during febrile attacks, %		Estimated %	Estimated %	N/A	
Gastrointestinal/abdominal		Onset	Cumulative		
Abdominal pain	85 %	20 %	63 %		88 %
Diarrhea	72 %	40 %	69 %		84 %
Vomiting	71 %	11 %	45 %		69 %
Hepatomegaly	22 %	25 %	37 %		N/A
Serositis	19 %	N/A	N/A		N/A
Pericarditis	N/A	0 %	4 %		N/A
Lymphoid tissue					
Lymphadenopathy	87 %	38 %	71 %		84 %^b^
Splenomegaly	32 %	32 %	63 %		N/A
Musculoskeletal					
Arthralgia	84 %	20 %	67 %		71 %
Arthritis	55 %	6 %	43 %		28 %
Myalgia	N/A	0 %	22 %		57 %
Cutaneous and mucocutaneous					
Skin lesions or maculopapular rash	69 %	43 %	67 %		39 %
Aphthous ulcers or stomatitis	49 %	15 %	43 %		60 %
Pharyngitis	N/A	N/A	N/A		28 %
General					
Cold chills	63 %	N/A	N/A		N/A
Headache	63 %	0 %	12 %		38 %
Malaise	N/A	N/A	N/A		65 %
Weight loss	N/A	N/A	N/A		66 %
Fatigue	N/A	N/A	N/A		63 %
Mood disorders	N/A	N/A	N/A		24 %
Thrombocytopenia	N/A	4 %	4 %		N/A
Macrophage activation syndrome	N/A	0 %	6 %		1 %
Associated long-term conditions, %				N/A	
AA amyloidosis	3 %	0 %			5 %
Abdominal adhesions	10 %	6 %			N/A
Joint contractures	4 %	N/A			N/A
Recurrent and/or severe infections	N/A	27 %			N/A
Severe pneumococcal infections	1 %	6 %			N/A
Hypogammaglobulinemia	N/A	6 %			N/A
Renal angiomyolipoma	N/A	6 %			N/A
Cerebellar syndrome	N/A	N/A			3 %
Seizures	N/A	N/A			5 %
Mental retardation	N/A	2 %			4 %
Chronic neurologic, abdominal, renal, pulmonary, endocrine, cutaneous, ocular, or hematologic involvement, erosive polyarthritis, and/or Sjögren’s syndrome	N/A	55 %			N/A
Biomarkers, median (range), % of the patients above the upper limit of the [normal value]				N/A	
WBC count, x10^9^/L during fever episodes [4–8]	15, ↑ in 100 % of the patients	18 (7.5–59)			↑ in 66 % of tested patients
CRP, mg/L during fever episodes [<5]	163 (36–404), ↑ in 100 % of the patients	157 (47–440), ↑ in 100 % of tested patients			↑ in 94 % of tested patients
ESR mm/hour during fever episodes [<10]	76, ↑ in 100 % of the patients	64 (27–120), ↑ in 100 % of tested patients			↑ in 98 % of tested patients
IgA, g/L [0.5–3.4]	4.1, 64 % of tested patients >2.6 g/L	4.8 (0.25–20.9), 57 % of tested patients >3 g/L			N/A
IgD, IU/mL [<100]	400 (<0.8-5300), ↑ in 78 % of the patients	760 (0–2500), ↑ in 88 % of tested patients			↑ in 72 % of tested patients
Urinary mevalonic acid, mmol/mol creatinine during fever episodes [<1]	N/A	17 (2.8–10000), ↑ in 100 % of tested patients			↑ in 93 % of tested patients
Biomarkers, median (range), % of the patients below the limit of the [normal value]					
MVK activity, % of control cells [>25]	N/A	2.6 (0–24), ↓ in 100 % of tested patients		N/A	N/A

Abbreviations: *CRP* C-reactive protein, *ESR* erythrocyte sedimentation rate, *HIDS* hyperimmunoglobulinemia D syndrome, *IgA* immunoglobulin A, *IgD* immunoglobulin D, *MKD* mevalonate kinase deficiency, *MVK* mevalonate kinase, *N/A* data not available, *WBC* white blood cell

^a^From the Eurofever registry by Toplak et al. [10] with 104 MKD patients

^b^From the Eurofever registry by Ter Haar et al. [5] with 85 MKD patients

### Genetic mutations and genotype-phenotype relationship

To date, 204 MVK sequence variants have been submitted to the Infevers, an online registry of hereditary autoinflammatory disorders mutations [19–21]. Most patients with MKD are compound heterozygotes for missense mutations [2, 22]. Other mutations include nonsense mutations, deletions, insertions, splicing defects, and a combination of a deletion and an insertion [2, 23]. The p.Val377Ile mutation was the most common MVK gene mutation with approximately 50 % of allele frequency among patients [2, 5, 8, 9, 22] (Table 1). The p.Val377Ile mutation was found to be present only in the HIDS phenotype in an earlier study [23]. However, in a more recent French and Belgian study, 6 patients homozygous for p.Val377Ile had variable severities of MKD ranging from asymptomatic, to mild, to severe [2]. The p.Ile268Thr mutation was the second most frequent MVK gene mutation with approximately 8–15 % of allele frequency among patients [2, 5, 8, 22, 23] (Table 1). The p.Ile268Thr mutation was found to be present in both HIDS and MVA phenotypes [23]. No relationship was observed between genotypes and the phenotype with respect to age at onset of symptoms, symptoms during febrile attacks, and the frequency of febrile attacks per year in the International HIDS Database [8, 24].

Mevalonate kinase is a key enzyme of the mevalonate pathway that produces isopentenyl pyrophosphate (IPP), downstream compounds of non-sterol isoprenoids such as farnesyl-pyrophosphate and geranylgeranyl-pyrophosphate, and cholesterol [1, 25]. The mutation in MVK results in reduced activity of mevalonate kinase, which in turn leads to decreased production of IPP and non-sterol isoprenoids and an accumulation of mevalonate [1]. IPP and non-sterol isoprenoids are important for various key physiological processes, including inflammatory pathways. Clinical manifestations of MKD have initially been hypothesized to be due to high levels of mevalonate. Recent data indicate that reduced availability of non-sterol isoprenoids play a critical role in the inflammatory phenotype of MKD, which is at least in part mediated through interleukin (IL)-1β [1].

### Clinical features and progression of disease

While the average age at onset of symptoms of MKD is within the first year of life, and before age 5 for the vast majority of the patients (2), it could vary from the first week of life to 20 years [2, 8–10] (Table 1). However, the median age at diagnosis is about 8 to 10 years, suggesting a delay by several years between symptom onset and diagnosis. This can be in part attributable to the initial misdiagnosis due to its rarity and the nonspecific nature of the clinical disease manifestations [8, 10, 26]. In the International HIDS Database, 33 of the 103 patients were offered an alternative diagnosis (Familial Mediterranean Fever [13 patients], adult-onset Still disease [6 patients], juvenile chronic arthritis [5 patients], rheumatic fever [3 patients], chronic infection [3 patients], and Behçet disease [3 patients]) before the diagnosis of HIDS [8]. Similarly, many diseases had been considered before the right diagnosis of MKD in a recent French study of 13 patients [26]. MKD appeared to affect both men and women equally [1, 2, 8–10, 17].

MKD is characterized by recurrent inflammatory attacks, with abrupt onset of high fever (frequently exceeding 40 °C) as the most noted symptom, and each episode lasts 3 to 7 days in most patients [1, 5]. Fever episodes occur spontaneously but also can be precipitated by vaccination, infection, or physical and emotional stress [2, 5, 8, 17]. Fever episodes occur irregularly every 2 to 8 weeks in HIDS patients [1] and with greater frequencies in MVA patients than in HIDS patients, with the former (MVA) reporting as often as 25 episodes and the latter (HIDS) averaging 12 annually [9, 17, 27].

Febrile attacks are frequently accompanied by a variety of signs and symptoms, including gastrointestinal complaints such as abdominal pain, diarrhea and vomiting; lymphoid tissue symptoms such as lymphadenopathy (mainly in the cervical region) and splenomegaly; musculoskeletal symptoms such as arthralgia and arthritis (mainly in large peripheral joints) and myalgia; cutaneous and mucocutaneous symptoms such as maculopapular rash, aphthous ulcers (or stomatitis) and pharyngitis; and other symptoms such as headache, cold chills, malaise and fatigue [2, 8, 17] (Table 1). All HIDS patients who had splenomegaly also had lymphadenopathy concurrently in the International HIDS Database [8]. Hepatomegaly was reported in 22 % of HIDS patients in the International HIDS Database [8], and the frequency of hepatomegaly was 25–37 % in MKD patients in the French and Belgian study [2] (Table 1). Hepatosplenomegaly was reported in the majority of patients with MVA in a European cohort of 11 MVA children [27]. Rare cases of cholestatic or non-cholestatic hepatitis have also been described in a few children with MKD [28–33]. Rare instances of macrophage activation syndrome, a life-threatening complication characterized by high fever, pancytopenia and liver dysfunction, have been reported during febrile attacks [2, 17]. Additional clinical features of MVA, the more severe form of MKD, include intrauterine growth retardation, various congenital defects (cataracts, shortened limbs, and dysmorphic craniofacial features), failure to thrive, neurological involvement (psychomotor retardation, developmental delay, progressive cerebellar ataxia, and hypotonia) [1, 27].

Febrile attacks are most frequent in childhood and decrease with age [8, 9], while disease activity will continue to be present in most affected patients [2, 8, 9]. In the International HIDS Database [8], the percentage of HIDS patients with >6 febrile attacks per year was approximately 90 % in the first decade of life and 73 % in the second decade of life. After the age of 20 years, 50 % of the HIDS patients remained to have >6 attacks per year. Furthermore, the percentage of HIDS patients with >12 febrile attacks per year was 44 % in the first decade of life, 24 % in the second decade of life, and 18 % over the age of 20 years. However, no patients had a remission [8]. In the French and Belgian study, 14 (45 %) of the 31 surviving symptomatic patients followed up for >5 years turned into asymptomatic or showed only mildly active disease and had a significant reduction in the frequency of febrile attacks (disease activity score of 0 [inactive disease] or 1 [mild] by the physician’s global subjective assessment of disease activity) without long-term treatment [2]. However, the other 17 of the 31 surviving symptomatic patients (55 %) in this study continued to have highly active disease as demonstrated by frequent febrile attacks or continuous organ involvement (disease activity score of 2 [severe]) [2]. There was no significant difference in mean age at disease onset (8.2 vs 12.2 months) or the frequency of the p.Val377Ile mutation (70 % vs 57 %) between patients with the persistently high disease activity or fatal outcome and those with the decreased disease activity over time [2]. In the Italian study, the number of fever episodes per year decreased from 13.8 ± 5.4 at baseline to 8.8 ± 6.7 at the follow-up. While 12 patients (29 %) displayed a spontaneous improvement of the disease (a reduction of >30 % of fever episodes or no fever episodes in the last 6 months without any maintenance therapy) at the follow-up, 15 (36 %) showed no changes, and 7 (17 %) were exacerbated [9]. In multivariate analysis, female sex and the homozygous state for p.Val377Ile were found to be significantly associated with the spontaneous improvement of disease course in patients with MKD [9].

MKD, including milder HIDS phenotype, is also associated with long-term complications. Type AA amyloidosis (3 % [frequency]), joint contractures (4 %), and abdominal adhesions (10 %) were reported in patients with HIDS in the International HIDS Database [8]. All 3 patients who developed amyloidosis experienced recurrent febrile attacks for over 20 years prior to the manifestation of amyloidosis [8]. Type AA amyloidosis (5 %) was also found in patients with MKD in the Eurofever registry [17]. In the French and Belgian study, although type AA amyloidosis was not observed, among 49 symptomatic patients with MKD, 27 patients (55 %) had chronic neurologic, abdominal, renal, pulmonary, endocrine, cutaneous, ocular, or hematologic involvement, erosive polyarthritis, and/or Sjögren’s syndrome; 13 patients (27 %) had recurrent and/or severe infections including otitis, sinusitis, and pneumonitis with at least 3 severe pneumococcal infections (6 %); and 3 patients (6 %) developed renal angiomyolipoma [2].

### Biomarkers

During febrile attacks in patients with HIDS [8] or MKD [2], there is a strong acute-phase response, with substantially elevated concentrations of white blood cell (WBC) count, C-reactive protein (CRP), and erythrocyte sedimentation rate (ESR), especially CRP and ESR (Table 1). CRP, ESR and WBC count are nonspecific markers of systemic inflammation [34]. Between febrile attacks in patients with HIDS in the International HIDS Database, the concentrations of these inflammatory biomarkers decreased, but continued to be elevated in some patients [8]. Similarly, a study showed that pro-inflammatory cytokines such as IL-1β and IL-6 were significantly increased in MKD patients (*n* = 8) even during the non-acute phase as compared to healthy controls (*n* = 35) [35].

Most patients with HIDS have elevated concentrations of IgD and IgA (Table 1). Although an elevated IgD concentration may suggest a possible diagnosis of HIDS, it is not diagnostic as some patients do not have an elevated IgD concentration [2, 8, 17]. The percentage of the patients with abnormal values of IgD was 78 % in the International HIDS Database [8], 88 % in the French and Belgian study [2], and 72 % in the Eurofever registry [17]. The diagnostic value of an elevated IgD concentration in patients with recurrent fever and clinical signs suggestive of MKD was found to be suboptimal, with a sensitivity of 79 %, a specificity of 27 %, a positive predictive value of 50 %, and a negative predictive value of 58 % [36]. An elevated IgD concentration is often accompanied by an elevated IgA, but the elevation in IgA is also not present in some patients (64 % of tested patients above 2.6 g/L in the International HIDS Database [8] and 57 % of tested patients >3 g/L in the French and Belgian study [2]).

There is a residual mevalonate kinase activity varying from 1.8 % to 28 % in HIDS patients, but little to no residual activity in MVA patients [1, 27]. Conversely, there are mildly to moderately elevated urinary mevalonic acid concentrations in patients with HIDS, but very high concentrations in plasma and urine in patients with MVA [1, 2, 27]. In a European cohort of 11 MVA children, plasma and urinary mevalonic acid concentrations were reported to be correlated with the severity of the clinical disease among patients and during the course of remission and exacerbation within patients [27]. Thus, the mevalonic acid concentration is a good indicator of the severity of the disease. All 33 MKD patients tested during febrile attacks in the French and Belgian study [2] and 37 of the 40 patients tested (93 %) in the Eurofever registry [17] had elevated urinary mevalonic acid concentrations, suggesting that the mevalonic acid concentration is also a sensitive biomarker for the screening of MKD [2]. The diagnostic value of the urinary mevalonic acid measurement in patients with a clinical suspicion of MKD was evaluated in a retrospective analysis of data from a single center in the Netherlands [37]. The analysis found a sensitivity of 92 %, a specificity of 90 %, a positive predictive value of 71 %, and a negative predictive value of 98 %, further indicating that the urinary mevalonic acid measurement is a reasonable biomarker to screen patients for MVK genetic testing and enzyme assay [37].

### Prognosis and survival

HIDS, the less severe form of MKD, is not typically life-threatening and is not in general related to a decrease in life expectancy [38]. While mildly affected patients with MVA may have a normal life expectancy [38], the most severely affected patients with MVA can die in early childhood [27, 38]. In the International HIDS Database, there were three deaths during the follow-up, but the causes of death (suicide, cerebral hemorrhage, and pneumococcal sepsis) were considered to be unrelated to HIDS [8]. In the French and Belgian study, which included both the mild and severe phenotypes of MKD, three patients died of causes associated with MKD at age of 2 to 3 years – two patients died of multiple organ failure and one patient died of staphylococcal sepsis associated with macrophage activation syndrome [2]. In a European cohort of 11 MVA children, 4 patients died during recurrent crises at age of 6 months to 4 years, and two of them had exceedingly high plasma and urinary mevalonic acid concentrations [27], suggesting that the mevalonic acid measurement may also potentially serve as a prognostic biomarker.

### Health-related quality of life (HRQOL)

HIDS significantly impairs several aspects of patients’ and caregivers’ quality of life [8, 39–41], including physical role functioning, social functioning, emotion, and finance, with an adverse impact on patients’ daily activities, education, employment status, independence, and family life [8, 39, 41]. Due to its rarity and nonspecific clinical manifestations, a number of alternative diagnoses and medical referrals may occur before the MKD diagnosis [8, 10, 26], which further augment the burden of the disease on both the patient and patient’s family. Thus, early diagnosis and treatment are critical in improving HRQOL.

Living with HIDS is unpleasant. During inflammatory attacks, patients experience high fever, nausea (especially in children), and pain [8, 39] and are often bedridden during severe flares [39]. A survey of HIDS patients in the US, Europe, and Australia also revealed that HIDS adversely affected patients’ relationships and social lives through limiting their activities, and for caregivers and adult patients, inflammatory attacks and doctor appointments caused missed work and limited career options, resulting in financial dependency [39]. In the International HIDS Database, HIDS was also reported to interfere with the development of autonomy and social development during childhood probably due to the increased dependency on caregivers and the decreased participation in peer and school activities because of their disease. Additionally, HIDS was reported to delay their education in 46 % of the patients and to make the high school graduation unachievable in 17 %. Moreover, 35 % of the patients reported that HIDS contributed to their loss of job. Furthermore, 27 % of the patients were unemployed at the time of the study, which was much higher than the unemployment rate in the general Dutch population. The negative impact on HRQOL by HIDS was correlated with the number of inflammatory attacks. HIDS patients who had >6 inflammatory attacks per year had significantly lower scores for the domains of pain, physical role functioning, and general health perception than those who had 6 or less attacks per year in the International HIDS Database [8].

### Response to treatment

Currently, there are no therapies approved for MKD [1]. A number of treatments have been tried to treat and prevent febrile attacks in some patients [1, 2, 8, 18] (Table 2). In the Eurofever registry, nonsteroidal anti-inflammatory drugs (NSAIDs) and corticosteroids were the most frequently used medications (39 and 33 of the 67 patients [58 % and 49 %], respectively) and were primarily administered as on demand therapy [18]. NSAIDs were mostly taken during febrile attacks and relieved symptoms in most patients [5] and resulted in a complete response in 5 (13 %) and a partial response in 25 (64 %) in the Eurofever registry [18]. Corticosteroids given in high dosage can reduce the duration of febrile attacks [18]. Corticosteroids provided a complete response in 8 (24 %) and a partial response in 22 (67 %) in the Eurofever registry and a partial response in 35 (63 %), but no complete response in combined data of 187 patients from a literature review by the Eurofever registry investigators [18]. Anakinra, an IL-1 receptor antagonist, was used in 27 of the 67 patients (40 %) in the Eurofever registry and the response was complete in 6 (22 %) and partial in 18 (67 %) in the Eurofever registry and was complete in 12 (34 %) and partial in 16 (46 %) from the literature review [18]. Etanercept, a tumor necrosis factor alpha (TNF-α) inhibitor, was used in 17 of the 67 patients (25 %) in the Eurofever registry and induced a complete or partial response in 55–65 % of the patients who were treated [18]. Canakinumab (a fully human anti-IL-1β monoclonal antibody) and adalimumab (a TNF-α blocker), the newer biologic agents, also generated a complete or partial response in several patients [18, 42]. The results from a recent open-label, single treatment arm study of canakinumab in 9 patients with active HIDS also showed a reduction in the frequency, duration, signs and symptoms of acute febrile episodes and the normalization of inflammatory markers [43]. Further, a recent literature review of the treatment of HIDS with biologics in children reported that complete or partial responses were 90 % in 21 HIDS patients treated with anakinra, 50 % in 16 HIDS patients treated with etanercept, and 100 % in 5 patients treated with canakinumab [44]. However, colchicine, statins, antibiotics, thalidomide, and cyclosporine have not shown to be effective [2, 8, 18].

**Table 2:** Response to treatment in 67 patients with MKD from the Eurofever registry and in combined data of 187 patients from a literature review (19 papers) by the Eurofever registry investigators^a^

Medication	Data source	No. of patients	Treated patients		Complete response		Partial response		Failure	
			No.	%	No.	%	No.	%	No.	%
NSAIDs	Eurofever	67	39	58 %	5	13 %	25	64 %	9	23 %
Corticosteroids	Eurofever	67	33	49 %	8	24 %	22	67 %	3	9 %
	Literature	187	56	30 %	0	0 %	35	63 %	21	38 %
Colchicine	Eurofever	67	17	25 %	0	0 %	6	35 %	11	65 %
	Literature	187	60	32 %	1	2 %	11	18 %	48	80 %
Statins	Eurofever	67	11	16 %	0	0 %	3	27 %	8	73 %
	Literature	187	31	17 %	0	0 %	10	32 %	21	68 %
Anakinra	Eurofever	67	27	40 %	6	22 %	18	67 %	3	11 %
	Literature	187	35	19 %	12	34 %	16	46 %	7	20 %
Canakinumab	Eurofever	67	2	3 %	1	50 %	1	50 %	0	0 %
	Literature	187	3	2 %	2	67 %	1	33 %	0	0 %
Rilonacept	Eurofever	67	1	1 %	0	0 %	1	100 %	0	0 %
Etanercept	Eurofever	67	17	25 %	1	6 %	10	59 %	6	35 %
	Literature	187	27	14 %	6	22 %	9	33 %	12	44 %
Infliximab	Eurofever	67	1	1 %	0	0 %	0	0 %	1	100 %
Adalimumab	Eurofever	67	2	3 %	0	0 %	1	50 %	1	50 %
	Literature	187	3	2 %	1	33 %	1	33 %	1	33 %

Abbreviations: *MKD* mevalonate kinase deficiency, *NSAIDs* nonsteroidal anti-inflammatory drugs

^a^Adapted from ter Haar et al. [18]. Response to treatment was classified as complete remission, partial remission, failure or worsening. Complete remission was defined as no signs of active disease and the normalization of reported inflammatory markers, allowing for the persistence of sequelae. Some patients received more than one treatment

Since few randomized controlled trials have been conducted, the available data on response to treatment are mostly derived from retrospective registries, case series, and case reports [18]. Although there is lack of high-quality evidence, the existing data suggest that NSAIDs and corticosteroids on demand may confer some benefits for MKD patients and that patients with inadequately controlled MKD may benefit from IL-1 blockade or TNF-α inhibition [8, 18].

For severely affected patients who are resistant to all other therapies, allogeneic hematopoietic stem cell transplantation (HSCT) has been suggested as an option [1, 45]. Several patients had successfully undergone allogeneic HSCT, with a remission of systemic inflammation and an improvement of neurological symptoms [31, 46, 47].

With respect to side effects associated with the repeated use of biologics in patients with MKD, pain and inflammation at the injection site, bacterial pneumonia, and herpes zoster infection have been reported for anakinra, injection site reaction, recurrent pharyngitis, and transient hepatitis for canakinumab, and upper respiratory infections for etanercept [44]. Long-term corticosteroid use is associated with a reduction in bone mineral accretion and an increased risk of osteopenia in children [48]. HSCT is associated with the inherent transplantation-related risks such as graft-versus-host disease [1, 46, 49] and is suggested to be considered only in severely affected patients resistant to all other therapies [1, 45].

## Conclusions

Symptoms of MKD, an ‘ultra-rare’ autosomal recessive autoinflammatory disease, usually begin within the first year of life, but there is a delay by several years in diagnosis. Recurrent febrile attacks are most frequent in childhood and decline with age. MKD impairs HRQOL of patients and their families with a negative impact on patients’ daily activities, education, and employment. MKD is associated with serious long-term complications. Although MKD manifested as HIDS in general does not reduce life expectancy, MVA can be life-threatening and cause death in early childhood. Currently, there are no treatments approved for MKD. Off-label use of anti-IL-1 and anti-TNF-α agents and corticosteroids appears to provide some but insufficient benefits. There are concerns on side effects associated with the repeated use of these drugs, especially in children [44]. The development of therapies for MKD is thus needed.

## Abbreviations

CRP: C-reactive protein
ESR: erythrocyte sedimentation rate
HIDS: hyperimmunoglobulinemia D syndrome
HRQOL: health-related quality of life
HSCT: hematopoietic stem cell transplantation
IgA: immunoglobulin A
IgD: immunoglobulin D
IL: interleukin
IPP: isopentenyl pyrophosphate
MKD: mevalonate kinase deficiency
MVA: mevalonic aciduria
MVK: mevalonate kinase
N/A: data not available
NSAIDs: nonsteroidal anti-inflammatory drugs
TNF-α: tumor necrosis factor alpha
US: United States
WBC: white blood cell
